# The impact of rate and rhythm control strategies on quality of life for patients with atrial fibrillation: a protocol for a systematic review

**DOI:** 10.1186/s13643-023-02197-2

**Published:** 2023-03-21

**Authors:** Powsiga Uruthirakumar, Rajendra Surenthirakumaran, Tiffany E. Gooden, Gregory Y. H. Lip, G. Neil Thomas, David J. Moore, Krishnarajah Nirantharakumar, Balachandran Kumarendran, Kumaran Subaschandran, Shribavan Kaneshamoorthy, Vethanayagam Antony Sheron, Mahesan Guruparan, on behalf of the NIHR Global Health Research Group on Atrial Fibrillation Management, Ajini Arasalingam, Abi Beane, Isabela M. Bensenor, Peter Brocklehurst, Kar Keung Cheng, Itamar S. Santos, Wahbi El-Bouri, Mei Feng, Alessandra C. Goulart, Sheila Greenfield, Yutao Guo, Gustavo Gusso, Rashan Haniffa, Lindsey Humphreys, Kate Jolly, Sue Jowett, Emma Lancashire, Deirdre A. Lane, Xuewen Li, Yan-guang Li, Trudie Lobban, Paulo A. Lotufo, Semira Manseki-Holland, Rodrigo D. Olmos, Elisabete Paschoal, Paskaran Pirasanth, Uruthirakumar Powsiga, Carla Romagnolli, Alena Shantsila, Kanesamoorthy Shribavan, Isabelle Szmigin, Kumaran Subaschandren, Meihui Tai, Ana C. Varella, Hao Wang, Jingya Wang, Hui Zhang, Jiaoyue Zhong

**Affiliations:** 1 Department of Community and Family Medicine, Faculty of Medicine, University of Jaffna, Jaffna, Sri Lanka; 2 Institute of Applied Health Research, University of Birmingham, Birmingham, UK; 3 Liverpool Centre for Cardiovascular Science at University of Liverpool, Liverpool John Moores University and Liverpool Heart & Chest Hospital, Liverpool, UK; 4 Department of Clinical Medicine, Aalborg University, Aalborg, Denmark; 5 Department of Cardiology, Teaching Hospital, Jaffna, Sri Lanka

**Keywords:** Atrial fibrillation, Quality of life, Rate control, Rhythm control, Systematic review, Protocol

## Abstract

**Background:**

Atrial fibrillation (AF) is the most common heart arrhythmia globally and it adversely affects the quality of life (QoL). Available rate and rhythm control strategies equally reduce mortality but may impact QoL differently. A number of systematic reviews have focused on the impact of specific strategies on QoL, though a 2006 review synthesized the evidence on the effect of all strategies on QoL, allowing for a clinically important comparison between the types of strategies. Many trials have been published since the review undertook the search in 2005; therefore, an update is needed. This systematic review aims to provide an update to the 2006 review on the impact of all rate and rhythm control strategies on QoL in people with AF.

**Methods:**

The following four databases and three clinical trial registries will be searched for primary studies: CENTRAL, MEDLINE, Embase, CINAHL, WHO International Clinical Trials Registry Platform, ClinicalTrials.gov, and ClinicalTrialsRegister.eu. No language restriction will be applied. The search will be limited to 2004 or later publication year to allow overlap with the search conducted by the 2006 review authors. Any randomized control trial that reports the QoL of adult (≥ 18 years) AF patients following an eligible rate or rhythm control intervention will be eligible for inclusion. Eligible interventions (and comparators) include pacing, atrioventricular node junction and bundle of HIS ablation, pharmacological therapy, radio frequency catheter ablation, cryoablation, pulmonary vein isolation, maze operation, pace maker implantation, and defibrillator implantation. Two reviewers will independently screen for eligible studies, extract the data using a piloted tool, and assess bias by QoL outcome using the RoB 2 tool. The suitability of conducting a meta-analysis will be assessed by the clinical and methodology similarities of included studies. If it is feasible, standardized mean differences will be pooled using a random-effects model and assessed appropriately.

**Discussion:**

The findings from this review will allow for meaningful comparisons between various rate and rhythm control strategies regarding their impact on QoL. This review will be useful for a wide range of stakeholders and will be crucial for optimizing the overall wellbeing of AF patients.

**Systematic review registration:**

PROSPERO CRD42021290542

**Supplementary Information:**

The online version contains supplementary material available at 10.1186/s13643-023-02197-2.

## Background

Atrial fibrillation (AF) is the most common cardiac rhythm abnormality worldwide and can cause substantial morbidity and mortality [1, 2]. AF is strongly associated with stroke and additional cardiovascular diseases such as coronary artery disease, valvular heart disease, heart failure, and hypertension [3]. With the global burden of AF expected to increase [4], it is not only imperative to study the clinical consequences of AF, but to also understand the personal impact of the various treatment regimens. People with AF often experience a number of symptoms which may impact other aspects of their health and wellbeing, especially when day-to-day activities become difficult or impossible to do. Additionally, people with AF taking blood thinners for treatment may be fearful of bleeding and this may impact on their willingness to take part in certain activities [5]. For these reasons, living with AF has been found to lower a person’s quality of life (QoL) [6]. Fortunately, many procedures and medications exist that control the heart rate or rhythm in AF patients [7]. Both rate and rhythm control strategies improve symptoms and neither are superior to improving survival [8]; however, the impact of these strategies on QoL may differ.

QoL is important for AF care, during treatment and in the evaluation of new therapies. The World Health Organization (WHO) defines QoL as “an individual’s perception of their position in life in the context of the culture and value systems in which they live and in relation to their goals, expectations, standards and concern” [9]. In addition, QoL is related to wellness resulting from a combination of physical, functional, emotional, and social factors [10]. Having a lower QoL can have a detrimental impact on patients living with chronic conditions, such as AF.

There are a number of existing systematic reviews on AF and QoL; however, most have focused on one particular control strategy instead of comprehensively reviewing all existing strategies [2, 11–13]. Thrall et al. evaluated the effects of all rate and rhythm control strategies on QoL, though several studies have been published since their search was carried out in January 2005 [11]. Thus, an updated review is needed. This systematic review will provide an update to the aforementioned review, with the aim to systematically assess studies published to determine the current impact of rate and rhythm control strategies on QoL in people with AF.

## Methods

This protocol is registered with the International Prospective Register of Systematic Reviews (PROSPERO) database (reference number CRD42021290542) [14]. We report this protocol in line with the Preferred Reporting Items for Systematic Reviews and Meta-Analyses Protocols (PRISMA-P) (see Additional file 1) [15].

### Eligibility criteria

#### Study design

Any randomized control trial (RCT) reporting the QoL of AF patients following an eligible rate or rhythm control intervention will be eligible for inclusion.

#### Population

Studies that include adult patients aged 18 years or older with AF will be considered eligible.

#### Interventions

The following rate and rhythm control strategies will be included: pacing, atrioventricular (AV) node junction and bundle of HIS ablation, pharmacological therapy, radio frequency catheter ablation, cryoablation, pulmonary vein (PV) isolation, maze operation, pace maker implantation, and defibrillator implantation.

#### Comparators

Any of the aforementioned interventions will also be considered as eligible comparators.

#### Outcome measures

QoL irrespective of the tool used will be the only eligible outcome. 

### Search strategy

The Cochrane Central Register of Controlled Trials (CENTRAL), MEDLINE, and Embase via OVID and CINAHL (Cumulative Index to Nursing and Allied Health Literature) will be searched for eligible studies. Additional file 2 contains an example of the search strategy in MEDLINE. The following trial registries will additionally be searched: WHO International Clinical Trials Registry Platform, ClinicalTrials.gov, and ClinicalTrialsRegister.eu. References of all included studies will be assessed for additional eligible studies not identified within the search.

The search strategy was developed using a combination of keywords and index terms. Key terms such as “atrial fibrillation,” “quality of life,” “rate control,” and “rhythm control” will be entered. There will be no restriction on language or setting. Publication date will be restricted to 2004 or later; this will allow for a 1-year overlap with the search date from the systematic review conducted by Thrall et al. (i.e., January 2005) [11] to ensure we include any studies missed in the 2006 published review due to delays in adding to databases or indexing within them. Studies that are included in the 2006 review will be included in our review if they meet the eligibility criteria.

Citations of all identified studies from the search strategy will be exported to Rayyan [16]. Duplicate citations will be removed using the automated features of Rayyan. Titles and abstracts will be assessed independently by two reviewers (PU and SK). The same two reviewers will independently assess the full text of any primary study that potentially meets the eligibility criteria. Any disagreements will be handled through discussion or a third reviewer when necessary (AS).

### Data extraction and management

Relevant data will be extracted from included studies using a piloted extraction tool in Excel. Data will be extracted by two reviewers independently (PU and SK); the data will then be cross-checked for accuracy.

The following information will be recorded:

- Study characteristics (title, authors, journal, publication date, study period, number of participants, country, any conflicts of interest, and funding source)
- Study design/methodology (study type, recruitment strategy, trial arm assignment strategy, eligibility criteria, QoL measurement tool, and duration of intervention)
- Study population characteristics (age, sex, type and duration of AF, and any comorbidities where available)
- Interventions (rate and rhythm control strategies) and comparator used
- Outcome (loss to follow-up, time points of assessment, QoL for intervention and control arms for each time point assessed; any results to statistical tests including any sub-group analyses)

We will contact the authors for any missing information. If there is no response after two attempts to contact, we will continue with the data available within the published article.

### Risk of bias assessment

Two reviewers (UP and SK) will independently assess the risk of bias using the Cochrane Collaboration Risk of Bias 2.0 (RoB 2) tool, providing a judgment of low risk of bias, high risk of bias, or some concerns for the QoL outcome from each included study [17]. Overall judgment of bias will be made by assessing each study’s randomization process, deviations from intended interventions, missing outcome data, measurement of the outcome, selection of the reported result, and any other potential process that could result in bias (for example, blinding). Any disagreements will be settled through discussion or by a third reviewer (AS) where necessary.

### Analysis plan

The study selection process will be summarized using a PRISMA-recommended study flow diagram [15]. A summary of findings table will present the characteristics of included studies along with reported outcome data relevant to our objectives.

Data will be managed in Excel and analyzed using Stata 14.0 (College Station, TX, USA). Continuous variables will be reported with mean and standard deviation and categorical variables with frequencies and percentages. Studies will be grouped by type of AF within the trial (paroxysmal, persistent, chronic, or permanent), intervention type, type of QoL assessment (generic or disease specific), and QoL tool used. The suitability of conducting a meta-analysis will be assessed by the clinical and methodological similarities of included studies. Any meta-analysis will use a pairwise random effects model due to the expectation that various tools will be used to assess QoL. If a meta-analysis is determined appropriate, the *I* ^2^ statistic and chi-squared test will be used to assess heterogeneity. If there is high heterogeneity (> 75%) and it appears to be from an outlier with an obvious bias (i.e., quality of study), a sensitivity analysis will be conducted (i.e., with low-quality studies excluded). A sub-group analysis for males and females will be conducted where feasible for rate and rhythm-control strategies separately. The effect sizes from each trial will be continuous and due to the expectation that different QoL tools will be used, standardized mean differences (SMDs) will be computed for any meta-analysis. Any pooled estimations of effect will be presented graphically as forest plots [18]. If there are 10 or more studies included in a meta-analysis, an assessment of the small study effect will be undertaken visually using a funnel plot. If a meta-analysis is not feasible, a qualitative synthesis of the results to each trial will be provided.

Level of certainty for the outcome of QoL for each included study and sub-group will be assessed using the Grading of Recommendation, Assessment, Development and Evaluation (GRADE) approach for systematic reviews. As per guidelines [19], we will critique the study limitations, inconsistency of results, indirectness of evidence, imprecision of findings, and other considerations that will contribute to the grading of the overall quality of evidence. The GRADE assessment results will be presented in the table of study characteristics as very low, low, moderate, or high. Explanations for any level other than high will be provided in the table footnotes.

## Discussion

Many rate and rhythm control strategies exist for people with AF and none is superior for survival [20]. However, understanding how each strategy impacts QoL is important for deciding which treatment options may be best for the patient’s overall wellbeing and in the study of new therapies for AF. A 2006 systematic review conducted by Thrall et al. found that AV node junction, bundle of HIS ablation, and pace procedures improved QoL in AF patients over a period of 6 to 12 months following the intervention [11]. When comparing rate versus rhythm control strategies, rate strategies often resulted in better improvements to QoL; however, the largest and only sufficiently powered trial found no difference [11]. Since the search was conducted in January 2005, many trials have been published on the impact of such strategies on QoL, though recent systematic reviews have only focused on particular strategies instead of reviewing the impact of all strategies on QoL [2, 12, 13]. Our updated review will provide important information on how the various rate and rhythm control strategies compare to one another with regard to QoL and whether the findings from Thrall et al. remain true.

This systematic review will provide a necessary update on the impact of rate and rhythm control strategies on QoL of people living with AF. There is one key limitation that may result from the methods of this systematic review. Some trials do not report QoL as a primary or secondary outcome and therefore may be missed from the search of the four databases given our QoL-related search terms, though we expect our search through three large trial registries will aid in reducing the risk of excluding eligible trials from the study. Furthermore, as this review will only include trials published over the last 15 years, we expect most trials to include QoL as an aim, as this becomes more common practice. The findings of this systematic review will be submitted for publication in a peer-reviewed journal and disseminated to stakeholders and the public. Within the manuscript, we will include the implications of our findings on clinical practice, policy, and future research. Therefore, our findings will be useful to a wide range of stakeholders. Publication of this research protocol is in keeping with good, transparent research practice, as it reduces the risk of bias and selective reporting while providing an opportunity to strengthen our proposed review. If the protocol is significantly revised in the future, we will document the amendments.

### Supplementary Information

**Additional file 1. **PRISMA-P checklist. Completed PRISMA-P checklist for this protocol.

**Additional file 2.** Search strategy for MEDLINE. An example of the search strategy, presented for MEDLINE.

## Data Availability

Not applicable.

## Abbreviations

AF: Atrial fibrillation
QoL: Quality of life
WHO: World Health Organization
PROSPERO: International Prospective Register of Systematic Reviews
PRISMA-P: Preferred Reporting Items for Systematic Reviews and Meta-Analysis Protocols
RCT: Randomized control trial
AV: Atrioventricular
PV: Pulmonary vein
CENTRAL: Cochrane Central Register of Controlled Trials
CINAHL: Cumulative Index to Nursing and Allied Health Literature
RoB 2: Risk of Bias 2.0
SMD: Standardized mean difference
GRADE: Grading of Recommendation, Assessment, Development and Evaluation
