# Correction: The impact of rate and rhythm control strategies on quality of life for patients with atrial fibrillation: a protocol for a systematic review

**DOI:** 10.1186/s13643-023-02434-8

**Published:** 2023-12-28

**Authors:** Powsiga Uruthirakumar, Rajendra Surenthirakumaran, Tiffany E. Gooden, Gregory Y. H. Lip, G. Neil Thomas, David J. Moore, Krishnarajah Nirantharakumar, Balachandran Kumarendran, Kumaran Subaschandran, Shribavan Kaneshamoorthy, Vethanayagam Antony Sheron, Mahesan Guruparan, the NIHR Global Health Research Group on Atrial Fibrillation Management

**Affiliations:** 1 Department of Community and Family Medicine, Faculty of Medicine, University of Jaffna, Jaffna, Sri Lanka; 2 Institute of Applied Health Research, University of Birmingham, Birmingham, UK; 3 Liverpool Centre for Cardiovascular Science at University of Liverpool, Liverpool John Moores University and Liverpool Heart & Chest Hospital, Liverpool, UK; 4 Department of Clinical Medicine, Aalborg University, Aalborg, Denmark; 5 Department of Cardiology, Teaching Hospital, Jaffna, Sri Lanka

**Correction: Syst Rev 12, 52 (2023)**

10.1186/s13643-023-02197-2 10.1186/s13643-023-02197-2

Following publication of the original article [1], the authors noticed that Additional file 2 was incorrect. The correct file is shown in this article and the file in the original article [1] was also corrected. The authors apologized for the inconvenience caused.

### Supplementary Information

**Additional file 2. **Search strategy for MEDLINE. An example of the search strategy, presented for MEDLINE.
