# Supplementary material for: The impact of rate and rhythm control strategies on quality of life for patients with atrial fibrillation: a protocol for a systematic review
Source: Syst Rev. 2023 Mar 21;12:52. doi: 10.1186/s13643-023-02197-2 (PMC10029179; doi:10.1186/s13643-023-02197-2)
Supplement: Supplementary file 2 — Additional file 2. Search strategy for MEDLINE. An example of the search strategy, presented for MEDLINE. [file 13643_2023_2197_MOESM2_ESM.docx]

Additional file 2: Search strategy for MEDLINE

Ovid MEDLINE(R) <1946 to August Week 4 2021>

1. exp Atrial Fibrillation/ or exp Arrhythmias, Cardiac/ (222252)

2. atrial fibrillation.ti.ab. or arrhythmia*.ti.ab. or AF.ti.ab. or Afib.ti.ab. (149889)

3. 1 or 2 (275260)

4. exp Quality of Life/ (225349)

5. quality of life.ti.ab. or well*being.ti.ab. or QoL.ti.ab. or HQoL.ti.ab. or HRQoL.ti.ab. (280668)

6. 4 or 5 (343325)

7. exp Ablation Techniques/ or exp Cardiac Catheterization/ or exp Pacemaker, Artificial/ or exp Electric Countershock/ or exp Anti-Arrhythmia Agents/ (424624)

8. ablation.ti.ab. or maze.ti.ab. or catheter*.ti.ab. or pace maker.ti.ab. or cardioversion.ti.ab. or rate control.ti.ab. or rhythm control.ti.ab. or anti*arrhythmia.ti.ab. or ventricular function.ti.ab. (327397)

9. 7 or 8 (675967)

10. 3 and 6 and 9 (1645)

11. limit 10 to yr=“2005-Current” (1160)
